# Human growth hormone (*GH1*) gene polymorphism map in a normal-statured adult population

**DOI:** 10.1111/j.1365-2265.2006.02718.x

**Published:** 2007-02-01

**Authors:** Cristina Esteban, Laura Audí, Antonio Carrascosa, Mónica Fernández-Cancio, Annalisa Pérez-Arroyo, Angels Ulied, Pilar Andaluz, Rosa Arjona, Marian Albisu, María Clemente, Miquel Gussinyé, Diego Yeste

**Affiliations:** Department of Pediatrics and Pediatric Endocrinology and Nutrition Research Unit, Hospital Materno-infantil Vall d’Hebron, Autonomous University of Barcelona Barcelona, Spain

## Abstract

**Objective:**

*GH1* gene presents a complex map of single nucleotide polymorphisms (SNPs) in the entire promoter, coding and noncoding regions. The aim of the study was to establish the complete map of *GH1* gene SNPs in our control normal population and to analyse its association with adult height.

**Design, subjects and measurements:**

A systematic *GH1* gene analysis was designed in a control population of 307 adults of both sexes with height normally distributed within normal range for the same population: −2 standard deviation scores (SDS) to +2 SDS. An analysis was performed on individual and combined genotype associations with adult height.

**Results:**

Twenty-five SNPs presented a frequency over 1%: 11 in the promoter (P1 to P11), three in the 5′UTR region (P12 to P14), one in exon 1 (P15), three in intron 1 (P16 to P18), two in intron 2 (P19 and P20), two in exon 4 (P21 and P22) and three in intron 4 (P23 to P25). Twenty-nine additional changes with frequencies under 1% were found in 29 subjects. P8, P19, P20 and P25 had not been previously described. P6, P12, P17 and P25 accounted for 6·2% of the variation in adult height (*P* = 0·0007) in this population with genotypes A/G at P6, G/G at P6 and A/G at P12 decreasing height SDS (−0·063 ± 0·031, −0·693 ± 0·350 and −0·489 ± 0·265, Mean ± SE) and genotypes A/T at P17 and T/G at P25 increasing height SDS (+1·094 ± 0·456 and +1·184 ± 0·432).

**Conclusions:**

This study established the *GH1* gene sequence variation map in a normal adult height control population confirming the high density of SNPs in a *relatively* small gene. Our study shows that the more frequent SNPs did not significantly contribute to height determination, while only one promoter and two intronic SNPs contributed significantly to it. Studies in larger populations will have to confirm the associations and *in vitro* functional studies will elucidate the mechanisms involved. Systematic *GH1* gene analysis in patients with growth delay and suspected GH deficiency/insufficiency will clarify whether different SNP frequencies and/or the presence of different sequence changes may be associated with phenotypes in them.

## Introduction

Human skeletal growth and final height attainment are a result of a multifactorial regulation involving systemic and local hormones, growth and nutritional factors, lifestyle and genetic factors. Heritability estimates^1^ and genome-wide linkage analysis^2^ have shown that genetic factors play a major role in determining stature. Among these factors, the GH-IGF-I axis plays an important role during postnatal life, and associations between structural variations in its genes and height are currently under study.^3^ Although growth hormone (GH) deficiency is a well-known cause of growth retardation, which responds to GH replacement therapy, the diagnosis and physiopathological mechanisms for the so-called ‘idiopathic isolated GH deficiency’ (IIGHD) require further clarification. In addition, GH secretion levels and markers of GH biological activity have been demonstrated to be specific and sensitive only in major deficiency states.^4^,^5^ Genetic causes of GH deficiency within the *GH1* gene have been established; however, they are rarely recognized and only sought in major GH deficiency states during childhood and in family studies.^3^ *GH1* gene, located at 17q22–24, is a component of the GH gene cluster in which five genes evolving from a common ancestor are 91–99% sequence conserved (paralogues).^6^ *GH1* is more abundantly expressed in pituitary cells, while the other four genes are expressed in placental tissue. Large deletions within the *GH1* gene cluster were described first followed by point mutations, the majority of which affect introns 3 or 4, provoke skipping of exon 3 product and exert a dominant effect.^3^,^7^,^8^ More recently, the presence of single nucleotide polymorphic points (SNPs) in the promoter region or in intron 4 of the *GH1* gene have been described^9^–^12^ and associations with promoter allele activities or with GH secretion efficacy and circulating IGF-I levels in growth-retarded patients have also been described.^11^,^12^ Other studies have analysed several *GH1* gene SNP genotypes as related to the incidence of neoplasia, with a positive association with colorectal neoplasia for intron 4 SNP,^13^ a negative result for breast carcinoma^14^,^15^ or a positive one for breast cancer risk.^16^,^17^ In addition, a recent study in a cohort of adults over ages 60 years detected a significant association between genotypes at one SNP in the *GH1* gene promoter region and at the intron 4 SNP described by Hasegawa *et al*.^11^ with baseline bone density and accelerated bone loss together with an interaction with weight at 1 year.^18^ Intron 4 SNP described by Hasegawa *et al*.^11^ has also been associated, in women, with shorter body height and reduced mortality,^19^ whereas another intron 4 SNP (T1169A) has been associated in both sexes with a favourable metabolic profile.^20^ A systematic SNP study was conducted by Adkins *et al*.^21^ in *GH1* promoter, coding and noncoding regions in DNAs from placental tissues, and analysis of associations between genotypes and birth weight revealed an association between an alternate nucleotide at −1 and +3 of translation initiation site and fetal growth restriction. However, no systematic *GH1* gene analysis in the entire promoter, coding and noncoding regions has been conducted in adults to establish the map of structural variation and its possible association with height. The relatively short size of the entire gene permits a complete analysis which is, nevertheless, hampered by the need to avoid amplification of any other of the GH cluster genes (paralogues) and the high density of sequence variations.

To obtain normative data for subsequent analysis of *GH1* gene contribution to IIGHD in children, a systematic *GH1* gene structural analysis was designed in a normal adult control population to establish the *GH1* gene SNP map in adults from our population with heights within the normal range, determine the genotype frequencies and analyse possible associations between individual and combined SNPs with height.

## Subjects and methods

### Subjects

A total of 307 adult subjects of both sexes (164 women and 143 men) were recruited from hospital personnel and parents of patients with no history of growth retardation. Subjects had to fulfil the following criteria: Iberian Peninsular (except Basque) family origin and no family history of pathological short stature. A single subject per family was included. The protocol was approved by the Hospital Vall d’Hebron Ethics Committee and written informed consent was obtained from each participant. Height standard deviation scores (height SDS) were calculated according to sex-specific reference growth charts for the Spanish population (Carrascosa *et al*.^22^ charts were used for subjects under ages 30 years and Hernández *et al*.^23^ for subjects aged 30–50 years). Only individuals with height SDS between −2 and +2 SDS were included in the study (mean −0·016; 32 women and 28 men between −2·000 and −1·010; 99 women and 80 men between −1·000 and +0·910; 33 women and 35 men between +1·010 and +1·980) and sample size was adjusted for normal sex and height SDS distribution. Height and weight were recorded in the morning by a single observer. Height was measured with a Harpenden stadiometer. Four millilitres of peripheral venous blood were drawn into EDTA-containing tubes for molecular genetic analysis.

### Genomic DNA study

Genomic DNA was obtained from peripheral blood following the method described by Lahiri and Nurnberger.^24^ DNA was amplified by polymerase chain reaction (PCR) using a nested strategy. Briefly, 50 ng of genomic DNA were added to a 10 µl reaction mixture of 1 mm Mg(OAc)_2_, 0·6 mm dNTPs, 0·3 µm of each primer, and 0·4 U *r Tth* DNA polymerase XL (Applied Biosystems, Foster City, CA, USA). The sense and antisense primers used corresponded to nucleotides 4156–5′ACGGTCCGCCACTACGCCCAGC-3′ and the complement of 6948–5′TGCAGTGAGCCAAGATTGTGCC-3′ of the GH gene cluster.^6^ The PCR reaction mix was denatured for 5 min at 94 °C and cycled 40 times (94 °C, 1 min; 72 °C, 3 min 30 s) followed by a 7-min extension at 72 °C. The resulting *GH1* PCR products (2893 bp) were used as templates for five nested reactions (AN, BL, CK, DI, FP), carried out as follows: 1 µl of each *GH1* PCR product was added to a 20 µl reaction mixture of 1·5 mm MgCl_2_, 0·2 mm dNTPs, 0·3 µm of each primer and 0·4 U Eco Taq DNA polymerase (Ecogen S.R.L., Barcelona, Spain). Reaction mixtures were denatured for 5 min at 94 °C, cycled 40 times (94 °C, 1 min; 58 °C, 1 min; and 72 °C, 1 min), followed by a 7-min extension at 72 °C. Sense and antisense primers were as follows:

sense A: 4573–5′AAGGGGAGAGCAAAGTGTGG3′;
antisense N: 5068–5′TGACGGGCTTGTGCTAAT3′;
sense B: 4983–5′GGGAAGGGAAAGATGACAAG3′;
antisense L: 5583–5′GGATAAGGGAATGGTTGG3′;
sense C: 5469–5′TCTGTTGCCCTCTGGTTTC3′;
antisense K: 6157–5′GGCGAAGACACTCCTGAG3′;
sense D: 5843–5′TGGTGGGCGGTCCTTCTC3′;
antisense I: 6601–5′AGCCCGTAGTTCTTGAGTAG3′;
sense F: 6229–5′AACGCTGATGGGGGTGAGG3′;
antisense P: 6748–5′GGTGGGCACTGGAGTGGCAA3′;

Sequencing from both ends was performed by the dideoxy method using ABI PRISM BigDye Terminator version 3·1 Cycle Sequencing Kit (Applied Biosystems, Foster City, CA, USA). *GH1* gene nucleotide sequence published by Chen *et al*.^6^ was used as control. For each DNA, the five segments from the nested PCR were assembled with the SeqEscape programme (Applied Biosystems) and interpretation was made visually and simultaneously by two observers. Antisense sequencing was performed to confirm each nucleotide sequence change up to the establishment of the more frequent SNP map (frequency over 1%), whereas less frequent single or multiple nucleotide changes were reconfirmed in each DNA by antisense sequence and resequencing after a new nested PCR from original DNA was performed.

### Single nucleotide polymorphism (SNP) genotyping

The sequences for the five genes of the GH cluster identified by Chen *et al*.^6^ and reported as the GI sequence 183148 were aligned using the Multalin program.^25^ SNPs and other sequence changes identified were indicated using their position corresponding to *GH1*. Genotypes were deduced by the combination of genetic variation at the polymorphic positions.

### Statistical analysis

Standardized height was investigated for normal distribution (Kolmogorov-Smirnov test: *c*^2^ = 2·882, *P* = 0·4733). Hardy–Weinberg equilibrium was tested for SNPs presenting three alternate genotypes according to standard procedures using χ^2^-analysis. anova test was applied to investigate individual and combined SNP association with adult height SDS; significance assessment was adjusted for multiple testing using Fisher's PLSD test setting *P*_critical_ = 0·05 or the Bonferroni–Dunn test setting *P*_critical_ = 0·05/*n* (*n* = number of comparisons carried out). Stepwise regression analysis was applied to predict the contribution of SNPs to adult height SDS. Statview 4·5 program (Abacus Concepts Inc., Berkeley, CA, USA) was used for statistical analyses.

## Results

### *GH1* gene sequence variation

*GH1* gene sequence comparison with the GI-183148 sequence published by Chen *et al.*^6^ yielded a total of 54 single or multiple nucleotide changes. Twenty-five SNPs presented a frequency over 1% (genotypes and frequencies are listed in Table 1). SNPs which presented the three alternate genotypes (P2 to P4, P6, P7, P10 and P24) were in Hardy–Weinberg equilibrium (data not shown). Twenty-nine additional changes were found with a frequency under 1% or involving more than one nucleotide and thus could be considered as rare variant SNPs (R1 to R29) (Table 2). These changes were found in 29 of 307 subjects (9·4%), all in heterozygosity.

**Table 1:** Frequencies of genotypes at SNPs in the *GH1* gene

				Percentage (%)		
				
Position†	Functional region‡	Location§	Nucleotide¶	Homozygous GI 183148	Heterozygous	Homozygous alternative
P1 = 4825*		Promoter	G-del	95·1	4·9	0
P2 = 4856*		Promoter	T-G	5·2	32·9	61·9
P3 = 4863*		Promoter	T-G	5·2	32·6	62·2
P4 = 4886		Promoter	G-T	31·6	50·8	17·6
P5 = 4996		Promoter	T-C	97·1	2·9	0
P6 = 5089	Pit 1p RE	Promoter	A-G	72·6	24·4	3·0
P7 = 5107*	VDR/RA/T3 RE	Promoter	G-T	39·7	48·9	11·4
P8 = 5116	VDR/RA/T3 RE	Promoter	G-A	98·7	1·3	0
P9 = 5131	TATA box	Promoter	G-del	84·1	14·0	1·9
P10 = 5157		Promoter	A-G	27·7	49·8	22·5
P11 = 5162		Promoter	A-T-**C**	81·4	16·9 (AT)/**0·9 (AC)**	0·6 (TT)
P12 = 5178		5′UTR	A-G	94·1	5·9	0
P13 = 5187		5′UTR	A-C	94·8	5·2	0
P14 = 5221*		5′UTR	G-T	0	6·5	93·5
P15 = 5231	Thr 3 Ala/Pro	Exon I	A-G-**C**	96·4	3·3 (AG)/**0·3 (AC)**	0
P16 = 5286		Intron I	A-G	98·1	1·9	0
P17 = 5290		Intron I	A-T	98·4	1·6	0
P18 = 5443		Intron I	T-C	94·5	5·5	0
P19 = 5681		Intron II	A-T	98·1	1·9	0
P20 = 5686		Intron II	G-A	98·3	1·7	0
P21 = 6191*	Val 110 Ile	Exon IV	G-A	98·4	1·6	0
P22 = 6232*	Thr 123	Exon IV	G-A	97·1	2·9	0
P23 = 6263*		Intron IV	C-T	95·8	3·9	0·3
P24 = 6331*		Intron IV	T-A	27·7	54·4	17·9
P25 = 6358		Intron IV	T-G	98·1	1·9	0

† SNPs are identified correlatively from P1 to P25 with corresponding numbering to Genebank accession GI 183148.

‡ Response elements previously reported in the *GH1* promoter region included in SNPs. Three SNPs are located in codons.

§ Gene region location.

¶ Nucleotides found at this position.

* In these positions, the less represented alleles do not correspond to any other paralogue of the *GH1* gene.

Allelic frequencies in bold: < 1% found in an SNP position.

**Table 2:** *GH1* additional sequence changes

Genotype	Position*	Location†	*GH1* paralogues‡	*n*§	Height SDS¶
1	**R1** = 4688 G > A	Promoter	*CS5*/*GH2*	2	1·080/1·520
2	**R2** = 4903 G > C + **R3** = 4907 C > A	Promoter	*CS5*	1	1·870
3	**R4** = 4979 C > T	Promoter	*GH2*/*CS1*/*CS2*	1	−1·780
4	**R5** = 5003 C > T + **R6** = 5078 T > G	Promoter	*GH2*	1	−1·100
5	**R7** = 5111–5125 CAGTGGGGAGAAGG > GCAGGGAGAGAGAACT	Promoter **(VDR/RA/T3 RE)**	*CS1*/*CS2*	1	0·640
6	**R8** = 5124–5125 GG > CT	Promoter **(VDR/RA/T3 RE)**	*CS1*/*CS2*	1	0·179
7	**R9** = 5122 delA + **R10** = 5123–5125 AGG > GAACT	Promoter **(VDR/RA/T3 RE)**	NO	1	0·200
8	5162 A > C (P11) + **R11** = 5165 G > C	5′UTR	*CS5*	2	−1·260/−0·40
9	5162 A > C (P11) + 5165 G > C (R11) + **R12** = 5266 T > C	5′UTR + Intron I	*CS5*	1	−0·510
10	**R13** = 5220 C > T + 5231 A > C (P15)	5′UTR + Exon I **(Thr3Pro)**	*CS1*	1	0·400
11	**R14** = 5300 G > T	Intron I	NO	1	−1·560
12	5300 G > T (R14) + (**R15** = 6056 A > C + **R16** = 6061 C > G)	Intron I + Intron III	NO + *GH2*/*CS1*/*CS2*/*CS5*	1	−1·930
13	**R17** = 5302 G > A	Intron I	NO	1	−0·300
14	**R18** = 5354 G > C + [6056 A > C (R15) + 6061 C > G (R16)]	Intron I + Intron III	*GH2*/*CS1*/*CS2*/*CS5*	1	0·060
15	**R19** = 5406 InsGA	Intron I	*GH2*/*CS1*/*CS2*/*CS5*	1	0·400
16	**R20** = 5481 T > C	Intron I	*CS1*/*CS2*/*CS5*	1	−0·750
17	**R21** = 5679 G > C	Intron II	NO	3	−0·690/ −0·160/1·350
18	6056 A > C (R15) + 6061 C > G (R16)	Intron III	*GH2*/*CS1*/*CS2*/*CS5*	1	0·057
19	**R22** = 6190 C > T	Exon IV **(Asn109)**	*CS1*/*CS2*/*CS5*	1	1·060
20	**R23** = 6344 C > T	Intron IV	NO	2	0·120/1·500
21	**R24** = 6453 A > G	Intron IV	*GH2*/*CS1*/*CS2*/*CS5*	1	−0·260
22	**R25** = 6514 C > A + **R26** = 6517G > T	Exon V **(Pro133Hys** **+ Arg134Leu)**	*CS5*	1	−0·026
23	**R27** = 6653 C > G	Exon V **(Ile179Met)**	*CS1*/*CS2*/*CS5*	1	−0·350
24	**R28** = 6671 G > A + **R29** = 6677 C > T	Exon V (**Val 185 + Gly 187**)	*CS2*	1	0·640

* Nucleotide changes found in less than 1% of controls with numbers corresponding to Genebank accession GI 183148.

† Gene region location: response elements, codons and predicted amino acids affected are highlighted.

‡ *GH1* gene paralogues corresponding to the nucleotide changes found. NO means that the nucleotide found does not correspond to any of the of *GH1* paralogues.

§ *n* = number of controls.

¶ Height SDS corresponding to each control bearing the sequence change.

### *GH1*-paralogue alignment

**Fig. 1:**
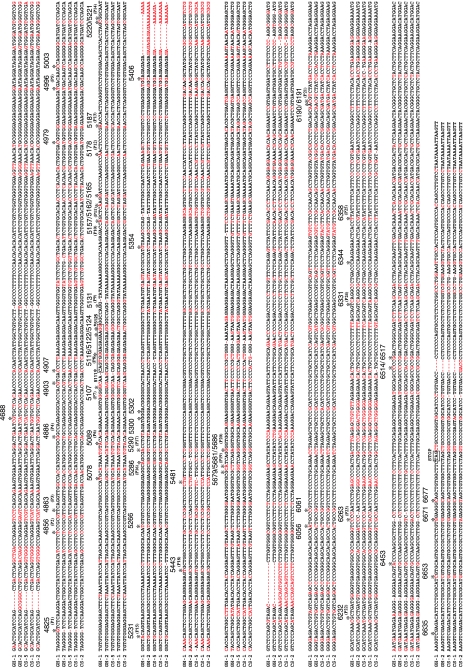
Alignment of GH gene cluster paralogues (*GH1*, *GH2*, *CS5*, *CS1* and *CS2*) from Genebank accession sequence GI 183148. The SNPs (P1 to P25) identified in the population, as well as 28 additional sequence changes, are signalled with their corresponding number. Segment of the *GH1* sequence translocated by *CS1*/*CS2* (box); segments where *GH1* and paralogues differ (in red); the STOP codon is marked.

A sequence alignment was performed to study possible sequence recombinations among paralogues of the five *GH1*-gene cluster (Fig. 1). This alignment showed that 9 of 25 SNPs (36%) in the *GH1* gene did not correspond to any of the paralogues.

Among the 29 rare SNPs found, six (20·7%) did not correspond to any of the paralogues: two were located in the 5′UTR region (R9 and R10), two in intron 1 (positions 5300 = R14 and 5302 = R17), one in intron 2 (position 5679 = R21) and one in intron 4 (position 6344 = R23) (Table 2).

### Equivalence with previously reported GH1 changes

Equivalence with changes and SNPs previously reported by other authors are shown in Table 3. The majority found a high density of SNPs in the promoter and 5′UTR regions in control populations.^10^,^12^,^21^ Several sequence changes have been reported in patients with familial or idiopathic short stature,^11^,^26^,^27^ whereas P8, P19, P20 and P25 (at positions 5165, 5681, 5686 and 6358, respectively, in the Genebank accession GI 183148) located in the promoter, intron 2 and intron 4 regions, respectively, had not been previously described.

**Table 3:** Location of *GH1* gene polymorphisms or mutations in previously reported and present study

Present study Position GI 183148	Wagner 1997 (10)	Giordano 1997 (9)	Hasegawa 2000 (11)	Horan 2003 (12)	Millar 2003 (26)	Lewis 2004 (27)	Adkins 2005 (21)	ENTREZ **SNP**
4688 (R1)				−476				
P1 = 4825	−400			−339				
P2 = 4856	−369			−308			−308	
P3 = 4863	−362			−301			−301	rs2011732
P4 = 4886	−339		P2	−278			−278	rs2005171
P5 = 4996	−229			−168			−168	rs2727338
P6 = 5089	−139	−75		−75			−75	rs11568828
P7 = 5107	−118	−57	P3	−57			−57	rs2005172
P8 = 5116								
5124–5125 (R8)					−40_−39 del GG ins CT			
P9 = 5131	−93/4			−31			−31	rs11568827
P10 = 5157	−68	−6		−6			−6	rs6171
P11 = 5162	−63	−1		−1			−1	rs695
5165 (R11)		+3		+3			+3	rs6175
P12 = 5178	−47	+16		+16			+16	rs9282699
P13 = 5187	−38	+25		+25			+25	rs6172
P14 = 5221	−4	+59		+59			+59	rs6173
P15 = 5231					Thr 3 Ala		+69	rs2001314
5266 (R12)								rs9282698
P16 = 5286							+124	
P17 = 5290							+128	
5300 (R14)	+76							
5302 (R17)							+140	
5354 (R18)								rs2001344
P18 = 5443	+219						+281	rs3744287
P19 = 5681								
P20 = 5686								
P21 = 6191					Val 110 Ile			rs5388
P24 = 6331			P1				+1070	rs2665802
P25 = 6358								
6653 (R27)						Ile179Met		

### *GH1* genotypes and associations with height SDS

Associations between genotypes and standardized height were first studied in the subpopulation of 278 controls carrying only the 25 most frequent SNPs in the *GH1* gene (*c*^2^ = 2·59; *P* = 0·5458 for normality of height distribution).

**Fig. 2:**
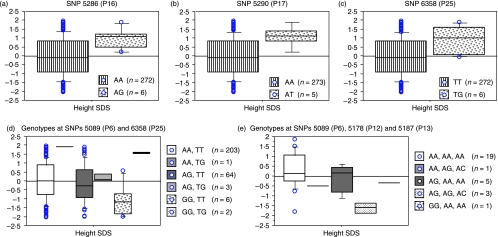
(a), (b), (c) Box plot distribution of height SDS in normal adult height population bearing the most frequent SNPs in the *GH1* gene (*n* = 278) according to genotypes in 5286 (P16), 5290 (P17) and 6358 (P25). SNPs P16 and P17 are in LD (*r*^2^ = 0·83). SNP P25 is carried in heterozygosis by six subjects homozygous at P16 and P17. Carriers of any of these polymorphisms in heterozygosis have significantly taller stature (*P* = 0·016 for P16, *P* = 0·015 for P17 and *P* = 0·023 for P25; Bonferroni–Dunn test). (d) Box plot distribution of height SDS in normal adult height population bearing the most frequent SNPs in the *GH1* gene (*n* = 278) according to their combined genotypes at SNPs 5089 (P6) and 6358 (P25). Subjects heterozygous T/G at P25 are taller than subjects with the same corresponding genotype at P6. Height SDS differed significantly between genotypes GG/TT and GG/TG (*P* = 0·0021; Bonferroni–Dunn test). (e) Box plot distribution of height SDS in normal adult height population bearing additional SNPs in the *GH1* gene with frequencies < 1% (*n* = 29) according to their genotypes at SNPs 5089 (P6), 5178 (P12) and 5187 (P13). Subjects carriers of heterozygous AG at P6 and/or P12 and P13 are significantly shorter than homozygous AA (*P* = 0·014; Bonferroni–Dunn test).

Three individual SNPs showed a statistically significant association with height SDS: at positions 5286 (P16), 5290 (P17) and 6358 (P25). Subjects with heterozygous genotypes presented statistically significant taller stature than the corresponding homozygous genotypes (*P =* 0·016 for P16, *P* = 0·015 for P17 and *P* = 0·023 for P25) (Fig. 2a,b,c). P16 and P17 were in linkage disequilibrium (LD) (*r*^2^ = 0·831), while P25 was carried by six subjects homozygous at P16 and P17.

*GH1* gene genotypes were defined by genetic variation in the 25 polymorphic positions. We found 163 different combinations. Only two genotypes presented a frequency over 5% (Table 4). Height SDS in the two more frequent genotypes did not differ significantly and covered the whole height range. Genotype 1 presented four heterozygous variations and Genotype 2 was the corresponding homozygous genotype. Heterozygous positions corresponded to SNPs with the highest frequency variation (4886 (P4), 5107 (P7), 5157 (P10) and 6331 (P24)). In addition, DNAs exhibited a different genotype in each of 129 subjects (46·4%).

**Table 4:** *GH1* more frequent genotypes in the control population bearing only 25 SNPs (*n* = 278)

*GH1* genotypes at the 25 SNPs							

	*GH1* gene region			Height SDS			
		
Genotype	Promoter region	5′UTR	Translatable region + Introns	Mean	SD	*n*	% (over 278)
1	GG/GG/GG/**GT**/TT/AA/**GT**/GG/GG/**AG**/AA	AA/AA/TT	AA/AA/AA/TT/AA/GG/GG/GG/CC /**TA**/TT	−0·006	1·148	22	7·9
2	GG/GG/GG/GG/TT/AA/TT/GG/GG/AA/AA	AA/AA/TT	AA/AA/AA/TT/AA/GG/GG/GG/ CC/TT/TT	0·043	1·031	21	7·5

*GH1* genotypes in the promoter region																	

SNP position in GI 183148												Height SDS					
	
Genotype	4825 (P1)	4856 (P2)	4863 (P3)	4886 (P4)	4996 (P5)	5089 (P6)	5107 (P7)	5116 (P8)	5131 (P9)	5157 (P10)	5162 (P11)	Mean	SD	Minimum	Maximum	*n*	% (over 278)
1	GG	GG	GG	**GT**	TT	AA	**GT**	GG	GG	**AG**	AA	−0·105	1·139	−2·000	1·980	35	12·6
2	GG	GG	GG	GG	TT	AA	TT	GG	GG	AA	AA	0·055	0·995	−1·760	1·480	25	9·0
3	GG	GG	GG	**GT**	TT	**AG**	**GT**	GG	GG	**AG**	AA	−0·495	0·835	−1·630	1·800	17	6·1
4	GG	**TG**	**TG**	GG	TT	AA	GT	GG	GG	AA	AA	0·008	1·070	−1·530	1·350	15	5·4
Combined single genotypes																53	19·1
Other combinations																133	47·8

*GH1* genotypes in the 5′UTR region									

SNP position in GI 183148				Height SDS					
			
Genotype	5178 (P12)	5187 (P13)	5221 (P14)	Mean	SD	Minimum	Maximum	*n*	% (over 278)
1	AA	AA	TT	−0·010	1·045	−2·000	1·980	250	89·9
2	AA	AA	GT	0·142	0·977	−1·180	1·350	14	5·1
3	AG	AC	TT	−0·177	1·145	−1·980	1·480	11	4·0
4	AG	AA	TT	−0·830	0·240	−1·000	−0·660	2	0·7
5	AG	AC	GT	−0·810				1	0·3

*GH1* genotypes defined by genetic variation at the 25 polymorphic positions present in > 1% of the population.

Genotype 1 presented four heterozygous positions (bold) corresponding to SNPs: P4, P7, P10 and P24 as shown in Table 1. These positions presented the highest allelic variation.

Genotype 2 was homozygous at all 25 SNPs.

Mean height SDS comparison between Genotypes 1 and 2 was not statistically significant.

*GH1* genotypes defined by genetic variation at 11 polymorphic positions in the promoter region in controls with 25 SNPs (*n* = 278).

Four genotypes had a frequency > 5%.

Heterozygous alleles (in bold).

Genotypes 1 and 2 corresponded to the more frequent complete *GH1* genotypes.

Genotype 3 differed from Genotype 1 in SNP at position 5089 (P6) (underlined).

Genotype 4 is heterozygous at positions 4856 (P2), 4863 (P3) and 5107 (P7).

*GH1* genotypes defined by genetic variation at the polymorphic positions in the 5′UTR.

SNPs at positions 5178 (P12) and 5187 (P13) were 85% associated. SNP at position 5221 (P14) was independent.

Mean height SDS comparison was not statistically significant.

The 11 SNPs found in the promoter region (Table 1) were grouped in 94 genotypes and analysed for association with adult height SDS. The four more frequent combinations are listed in Table 4: height SDS of these four genotypes did not differ statistically although Genotype 3 tended to have a shorter height. Genotype 3 differed from Genotype 1 in the SNP located at position 5089 (P6), corresponding to Pit 1 proximal responsive element for *GH1* gene promoter. Genotype 4 is heterozygous at positions 4856 (P2), 4863 (P3) and 5107 (P7). In addition, 19% of cases exhibited a genotype in the promoter region carried by only one subject.

Combination of the three SNPs in the 5′UTR region of *GH1* gene resulted in five different genotypes. SNPs at positions 5178 (P12) and 5187 (P13) were in LD (*r*^2^ = 0·88). Mean height SDS comparison among these genotypes was not statistically significant, although mean height SDS of alternate nucleotide carriers at position 5178 (P12) tended to be shorter (Table 4).

An anova analysis was conducted to investigate the interaction between two or more SNPs and height SDS. SNPs at positions 5286 (P16) and 5290 (P17) were in LD (*r*^2^ = 0·83): the heterozygous genotype AG/AT for these SNPs was associated with taller stature (shown above). SNP at position 6358 (P25) increased the expected height SDS for individual carriers of the G allele at 5089 (P6) SNP as shown in Fig. 2(d): subjects heterozygous at 6358 (P25) were taller than the mean, and mean height SDS of subjects with GG/TG combined genotype was significantly higher than the corresponding GG/TT genotype (*P* = 0·0021), suggesting an interaction between these two SNPs as they were not in LD.

Analysis of height SDS association with the most frequent single and combined SNPs and with rare variant SNPs was performed in the 29 individuals carrying the rare SNPs (Table 2). None of them carried any of the three SNPs (P16, P17 and P25) related to taller stature in the population of 278 controls with only the frequent SNPs. In these 29, mean height SDS (0·000 ± 0·987, from −1·930 to +1·870) did not differ from that of the 278 controls (−0·018 ± 1·041, from −2·000 to +1·980). Analysis of associations between individual SNP genotypes and height SDS revealed that SNPs at positions 5089 (P6), 5178 (P12) and 5187 (P13) were associated with significantly shorter stature (Fig. 2e). Only two sequence changes considered as rare SNPs were carried by individuals in the lower normal height range (between −1·500 and −2·000 SDS) (Table 2): R4 (4979 C > T) in the promoter region and R14 (5300 C > T) in intron 1. Predicted single amino acid changes located in exon 5 (R25 to R27) were not associated with short stature.

In the entire population of 307 controls, stepwise regression analysis between height SDS and genotypes at the 25 SNPs showed that genotypes at 5089 (P6), 5178 (P12), 5290 (P17) and 6358 (P25) were significantly correlated with height SDS (*r*^2^ = 0·062, *P* = 0·0007) with genotypes A/G at P6, G/G at P6 and A/G at P12 decreasing height SDS (−0·063 ± 0·031, −0·693 ± 0·350 and −0·489 ± 0·265, respectively, Mean ± SE) and genotypes A/T at P17 and T/G at P25 increasing height SDS (+ 1·094 ± 0·456 and +1·184 ± 0·432, respectively).

## Discussion

Genetic variations within human *GH1* gene have been described by several authors.^9^–^12^,^21^ The populations described to date comprised small numbers of normal-stature individuals,^9^ male adults with narrow height range^12^ or growth-retarded patients with/without GHD before achievement of adult height.^9^,^11^,^12^ Our study was designed to characterize the *GH1* gene sequence variation in individuals within the whole range of normal adult height (between −2 and +2 SDS) according to the standards for our population. Height was normally distributed, both sexes were equally represented and the GI-183148 homozygous sequence^6^ was used for comparison. A nested PCR with specific primers for *GH1* gene was designed, thus avoiding amplification of any other GH gene paralogue of the GH gene cluster.

Our results establish a map of 25 SNPs as present in over 1% of individuals, whereas 29 other sequence changes (single or multiple nucleotide) are present in less than 1% of subjects. More than 50% (*n* = 14) of SNPs are located in the promoter and 5′UTR regions, thus confirming previous reports: Giordano *et al*.^9^ reported eight SNPs in the promoter and 5′UTR regions, Wagner *et al*.^10^ 16 SNPs from the promoter to intron 1 and Horan *et al*.^12^ identified 36 haplotypes in control subjects of the British population, which would result from the combination of 15 of the previously reported SNPs. Our results confirm the presence of 13 of those points; SNP at 5165 (R11 in the present study and +3 in references^9^,^12^) was present in less than 1% of subjects and a new SNP is described (P8 at 5116 in the VDR/RA/T3 responsive element sequence). The remaining SNPs (from P15 to P25, *n* = 11) are distributed in introns 1, 2 and 4, and among coding regions only exon 1 and exon 4 bear a total of three SNPs, two of which predict an amino acid change (P15 and P21). These two latter SNPs had been described by Millar *et al*.^26^ and the more frequent SNP in intron 4 (P24) has been described by Hasegawa *et al*.^11^ together with the two more frequent SNPs in the promoter region (P4 and P7 in our map). Three new SNPs are described (P19 and P20 in intron 2 and P25 in intron 4), all outside the splice sites. Only SNPs presenting high frequency are present in homozygous alternate state and this accounts mostly for the majority of the promoter and 5′UTR SNPs and in the intron 4 more frequent SNP (P24) described by Hasegawa *et al*.^11^ In conclusion, in the entire coding and noncoding *GH1* gene sequence, only P24 is present in homozygous alternate state. Our results show that the GI 183148 homozygous sequence is present in our population except for SNP P14 in the 5′UTR region which is only present as the alternate nucleotide in homozygous or heterozygous states.

As described by several authors^9^,^10^,^12^,^21^ several promoter SNPs affected functional sequences and P6 is located in the Pit 1 proximal responsive element, P7 and P8 in the VDR/RA/T3 responsive element and P9 (G del) in the TATA box.

The mechanisms by which the high density of SNPs in the *GH1* gene is generated has been proposed to be recombination and gene conversion with any other(s) of the GH cluster genes.^9^,^12^,^28^ Alignment of the 25 SNPs with the other *GH1* gene paralogues demonstrated in our results that this mechanism is possible for 64% of SNPs. Familial SNP transmission pattern analysis will be of interest to support the hypothesis of GH gene recombination.

In addition, 29 of 307 individuals (9·4%) bore additional *GH1* sequence changes with frequencies under 1%. As for SNPs, they are located along the whole gene with higher density in the promoter and 5′UTR regions. Interestingly, intron 3 and exon 5 present several of these less frequent changes. Intron 3 has been shown to carry the majority of single nucleotide mutations causing the dominant form of GH deficiency.^3^ The two single nucleotide changes detected in intron 3 (R15 at 6056 and R16 at 6061) are in perfect LD (*r*^2^ = 1·0) and located within the enhancer splice site element (ESE) described by Ryther *et al*.^29^,^30^ Studies in additional normal or growth-retarded populations will permit description of their possible clinical implications. Five single nucleotide changes are located in exon 5; of these five, three predict an amino acid change, and one of the three (Ile179Met) has been described by Lewis *et al*.^27^ in a paediatric patient with familial short stature and the other two, as yet undescribed, are contiguous in a single individual (Pro133Hys and Arg134Leu). Polynucleotide changes are mostly located in the promoter region corresponding to the VDR/RA/T3 response element. As for frequent SNPs, the majority of the sequence changes with frequencies under 1% may have been generated by recombination within the GH gene cluster as 19 of 24 (79%) may correspond to one or more of the *GH1* gene paralogues.

Our results now show the diversity and complexity of SNP genotypes, as previously highlighted by other authors^9^,^10^,^12^,^21^ in a normal adult height control population. Our initial aim when designing the present study was to establish the map of *GH1* gene SNPs in our adult control population with heights normally distributed within the entire normal range for further comparison with genotypes in our paediatric population with growth delay, variable response to GH secretion tests and adequate response to GH therapy. Analysis of SNP association with adult height was subsequently performed to establish a body of knowledge useful for comparing patient genotypes and phenotypes. This analysis was first performed in controls bearing only frequent SNPs (90·5% of the total population). We demonstrate that the four SNPs with the highest allelic variation frequencies (P4, P7, P10 and P24) do not significantly contribute to adult height determination, with the heterozygous genotype being the most frequent followed by the corresponding homozygous genotype in the whole sequence, and heights are normally distributed over the entire height range. The third most frequent combined genotype in the promoter region in our population presented, in addition, in heterozygosity, the SNP at P6 in the sequence regulated by Pit 1 and although mean height of individuals (6·1%) bearing this genotype was around −0·5 SDS, this was not statistically significant.

Analysis of single SNP genotype association with adult height yielded few clues as to the contribution of *GH1* gene variation to adult height determination. Only three SNPs (P16, P17 and P25), present with low frequency and only in heterozygous state, were individually significantly associated with taller stature and none was individually associated with shorter stature. P16 and P17 (in LD, *r*^2^ = 0·83) are located in intron 1 and P25 in intron 4. The resulting sequence for the presence of P16 and P17 corresponded to the paralogue *GH2* and generated a responsive element for a core-binding protein (Matinspector Programme, Geometrix Software GmbH, München, Germany) with three Kruppel-type zinc fingers which could increase the efficacy of *GH1* gene transcription;^31^,^32^ moreover, Kruppel-like proteins have recently been described in the brain.^33^ Stepwise regression analysis demonstrated that P17 and P25 contribute, separately, to an increase of almost 1·0 height SDS. P16 and P17 had been described by Adkins *et al*.^21^ although they found no association with fetal growth, whereas P25 had not previously been described. The mechanisms by which they may determine taller final height should be established by *in vitro* studies analysing *GH1* gene transcription and GH protein translation efficiencies.

Analysis of interaction effect between SNPs detected that variation at P25 masked an effect of P6. Individuals homozygous at P25 (TT) present a significant association between P6 genotype and height with the homozygous alternate genotype at P6 (GG) being associated with shorter stature. This was further confirmed in the subpopulation of 29 individuals bearing rare SNPs who, in the absence of heterozygous change at P25, presented significantly shorter stature in the heterozygous alternate nucleotide change at P6 (AG). P6, located at Pit 1 proximal responsive element of the *GH1* gene promoter, was first described by Wagner *et al*.^10^ and Giordano *et al*.^9^ and further by Horan *et al*.^12^ Six of nine *GH1* gene promoter haplotypes bearing the alternate G at P6 presented lower transcriptional activities and electrophoretic mobility shift assays (EMSA) detected differential protein binding strength, although *in vitro* studies were unable to identify this SNP as a major determinant of *GH1* gene expression level.^12^ A recent study from Giordano *et al*.^34^ has shown a twofold reduced luciferase activity for the G nucleotide bearing promoter haplotype in transfected rat pituitary cells. Genotypes at P6 had also been associated with decreased breast cancer risk through its association with lower GH secretion and IGF-I circulating levels.^16^,^17^

In our results, *GH1* gene polymorphic structural variation accounted for only 6·2% of adult height determination in the entire adult population studied and genome-wide linkage analysis of stature in multiple populations revealed no linkage with chromosome 17 GH gene cluster.^2^,^35^ As only some of the less frequent SNPs are statistically associated with height, and in view of the high density of SNPs, our study may be hampered by selection bias^36^ and would ideally have required a wider sampling of some 2 000 individuals; however, this was a highly laborious strategy when the complete sequencing technique is applied. The high density of SNPs and their proximity hamper other genotyping strategies for rapid determination of the complete *GH1* SNP map in large control and patient populations. Individual SNP associations with height or other GH secretion-related phenotypic traits will require further confirmation by studies in larger populations and by *in vitro* functional studies.

In conclusion, our study established the *GH1* gene sequence variation map in an adult control population with heights normally distributed within the normal range. SNPs and other sequence change contributions to skeletal growth as observed at adult height demonstrated that, despite the high frequency of variation and diversity and complexity of combinations, only some of the less frequent SNPs were associated with taller stature (P17 in intron 1 and P25 in intron 4), even masking the SNP contribution to a shorter one (P6 in the promoter and P12 in the 5′UTR regions, respectively). Systematic *GH1* gene analysis in patients with growth delay and suspected GH deficiency/insufficiency will clarify whether different SNP frequencies and/or the presence of different sequence changes may be associated with phenotypes in them.
